# siRNA-mediated inhibition of endogenous brain-derived neurotrophic factor gene modulates the biological behavior of HeLa cells

**DOI:** 10.3892/or.2021.7922

**Published:** 2021-01-05

**Authors:** Chun-Yan Sun, Zhang-Bo Chu, Jing Huang, Lei Chen, Jian Xu, Ao-Shuang Xu, Jun-Ying Li, Yu Hu

Oncol Rep 37: 2751-2760, 2017; DOI: 10.3892/or.2017.5569

Following the publication of this article, an interested reader drew to the authors attention that, in Fig. 7 on p. 2757, sections of the data panels in Fig. 7A and B, showing the results of the non-transfected HeLa cell (Pnon group) and pGenesil-1-transfected HeLa cell (P0 group) experiments respectively, were strikingly similar. Both the Pnon and the P0 groups were control groups; upon re-examining their original data, the authors have realized that, when uploading the images, the data shown in Fig. 7B for the pGenesil-1-transfected HeLa cells (P0 group) were selected incorrectly. The authors were able to locate the data that were intended to have been shown in Fig. 7B; moreover, the text describing the number of migrated cells in the Results section also requires a correction. In the ‘*Downregulation of BDNF expression suppresses the migratory and invasive capabilities of HeLa cells*’ subsection, the text on lines 9–11 of this paragraph should be changed to the following (changed text is highlighted in bold): ‘Migrated cells/field in the PBDNF1 group (37±17) were significantly less than those in the Pnon (105±31) and P0 (86±27) groups’. Likewise, the same correction to the text has been made to the Figure legend, as shown opposite.

The revised version of Fig. 7, showing the correct data for Fig. 7B, is shown opposite. The authors are grateful to the Editor of *Oncology Reports* for allowing them the opportunity to publish this Corrigendum, and all of the authors agree to the publication of this Corrigendum. The authors sincerely apologize for this mistake, and regret any inconvenience this mistake has caused.

## Figures and Tables

**Figure 7.**
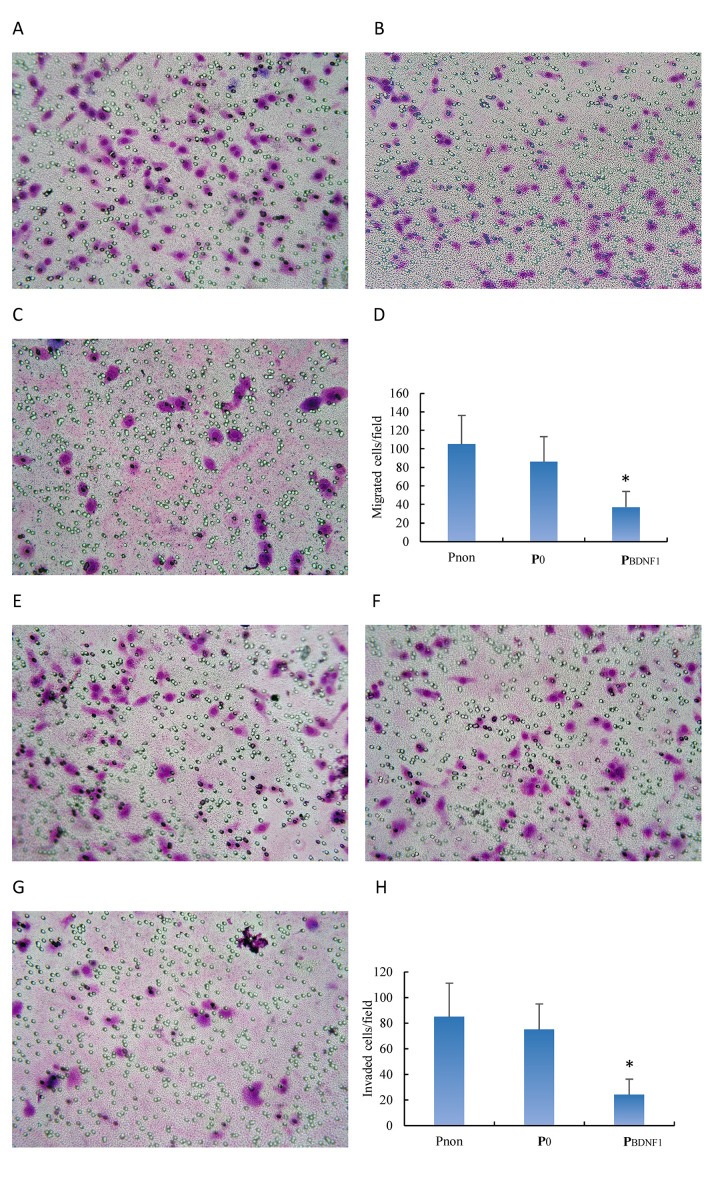
Downregulation of BDNF expression suppresses the migratory and invasive capabilities of HeLa cells. Migratory capabilities of cells in the Pnon, P0 and PBDNF1 groups were determined by Transwell assay. Cells (1×10^6^/ml) were plated in the upper chamber of the filters that had been coated with gelatin on the underside. At 6 h after plating, cells that had migrated to the underside of the filters were fixed and stained with Wright staining. Cells were counted at a magnification of ×400 using standard microscopy, and the mean number of cells/field in 5 random fields was recorded. (A-C) Migrated cells/field in the (C) PBDNF1 group (37±17) were significantly less than those in the (A) Pnon (105±31) and (B) P0 group (**86±27**) (*p<0.01). (E-G) Invasion assay was performed as above except for the upper surface of filters were coated with 25 µg Matrigel, the cell concentration was adjusted to 2×10^5^ cells/ml and the time was prolonged to 24 h. Invaded cells/field in the (G) PBDNF1 group (24±12) were significantly less than those in the (E) Pnon (85±26) and (F) P0 (75±20) (*p<0.01) group. Graphical illusion of the number of migrated cells is shown in (D) while invaded cells are shown in (H). Data are shown as the mean ± SD of 3 independent experiments.

